# Hyperuricaemia and its association with other risk factors for cardiovascular diseases: A population‐based study

**DOI:** 10.1002/edm2.387

**Published:** 2022-10-20

**Authors:** Farzaneh Yazdi, Mohammad Reza Shakibi, Amir Baniasad, Mohammad Javad Najafzadeh, Hamid Najafipour, Fatemeh Yazdi, Samira Sistani

**Affiliations:** ^1^ Physiology Research Center, Institute of Neuropharmacology Kerman University of Medical Sciences Kerman Iran; ^2^ Endocrinology and Metabolism Research Center, Institute of Basic and Clinical Physiology Science Kerman University of Medical Sciences Kerman Iran; ^3^ Resident of Internal Medicine, Endocrinology and Metabolism Research Center, Institute of Basic and Clinical Physiology Science Kerman University of Medical Sciences Kerman Iran; ^4^ Medical Intern, Student Research Committee Kerman University of Medical Sciences Kerman Iran; ^5^ Cardiovascular Research Center, Institute of Basic and Clinical Physiology Sciences Kerman University of Medical Sciences Kerman Iran; ^6^ Resident of Pediatrics, Endocrinology and Metabolism Research Center, Institute of Basic and Clinical Physiology Science Kerman University of Medical Sciences Kerman Iran

**Keywords:** diabetes, hypertension, hyperuricaemia, obesity, overweight, physical activity

## Abstract

**Introduction:**

Cardiovascular diseases are very common in the general population, and several factors play a role in their development. The purpose of this study was to investigate the relationship between hyperuricaemia and other cardiovascular disease risk factors.

**Methods:**

This cross‐sectional study was conducted on 1008 people over the 15‐year‐old general population in Kerman, Iran. The blood samples of all patients were analysed for the uric acid serum level, and they completed a checklist including physical activity, previous history of hypertension and diabetes, smoking and opium.

**Results:**

A number of 1008 cases of people were entered into the study. According to the results of this study, 254 patients had uric acid levels above the 75th percentile (6 mg/dl in males, and 5 mg/dl in females). No significant difference was observed between gender (*p* = .249) and age groups (*p* = .125) of people with and without hyperuricaemia. The prevalence of overweight/obesity (*p* < .001), hypertension (*p* = .004) and low physical activity (*p* = .033) was significantly higher in patients with hyperuricaemia. The duration of hypertension was significantly higher in hyperuricaemic individuals (*p* = .022). Overweight/obesity (OR = 2.67; 95% CI = 1.87–3.82) and hypertension (OR = 1.40; 95% CI = 1.02–1.93) were two significant independent factors that contributed to the increased risk of hyperuricaemia in the subjects.

**Conclusion:**

The uric acid serum level is higher in people with hypertension and overweight/obesity. Hyperuricaemia increases the risk of cardiovascular events, which can be prevented by determining the appropriate strategy for the early diagnosis and treatment of this metabolic disorder.

## Graphical abstract

Our study showed that the risk of elevated uric acid levels is higher in people with hypertension and overweight/obesity. Since hyperuricemia is one of the influential factors in the occurrence of cardiovascular events, these events can be prevented by determining the appropriate strategy for early diagnosis and treatment of this metabolic disorder.

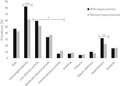

## 1 INTRODUCTION

Cardiovascular diseases are one of the most important causes of death in the world, and their pathogenesis is such that they are affected by several risk factors such as obesity, diabetes and hypertension.^1^ Increased serum uric acid levels can lead to endothelial dysfunction resulting in atherosclerosis by releasing reactive oxygen species and is reported as one of the risk factors for cardiovascular diseases.^2^

In the process of purine decomposition, uric acid is the final product.^3^ A condition in which serum uric acid concentration is high but there are no signs or symptoms of uric acid deposition is called asymptomatic hyperuricaemia. Although gout can occur in a person with any rate of uric acid, about two‐thirds of hyperuricaemic people remain asymptomatic.^4^ Different studies in different races have had different results on the prevalence of hyperuricaemia in people's age and gender.^5^, ^6^

Prevalence of hyperuricaemia is different in viewpoints of race, age and gender groups, and this serum factor has been mentioned as a risk factor for coronary artery diseases; in this study, the relationship between cardiovascular risk factors and hyperuricaemia was investigated.

## 2 MATERIALS AND METHODS

This study was a sub‐analyse of collected data about the risk factors of coronary artery diseases (i.e., Kerman Coronary Artery Disease Risk Factor Study; KERCADRS). This cross‐sectional and descriptive study was conducted in Kerman city on 1008 people who were over 15 years old. The sampling method of the studied people was in the form of a cluster from the entire population of the city. These people were selected from 720,000 Kerman residents, which is the biggest city in Iran's southeast, and they are the representative of this population. In the first phase of the KERCADRS, 250 postal codes (called seeds) were randomly selected from the list of city residents registered in the city post office. First, the location of each seed was determined on the map, and then, each of them was contacted. After identifying the number of people in each family, people over 15 years old were invited to participate in the study. If people do not answer despite calling twice, their neighbour was replacing them based on their seed address. In the first phase of the KERCADRS study, the collection of samples continued until 24 people was collected in each cluster. In the second phase of this study, people were called again, and 1008 people who met the criteria for entering our study were included.

The study protocol was reviewed and approved by the ethics committee of Kerman University of Medical Sciences (ethic code: IR.KMU.REC.1394.59). All study participants gave informed consent. The method of data collection has already been published in the study of Najafipour et al.^7^

Some risk factors, including gender, age, physical activity, smoking status, using tobacco products, body mass index (BMI), opium addiction and history of hypertension or diabetes, the duration of hypertension, and duration of diabetes disease, were reviewed in our study.

People's height was measured in a standing position without shoes. All participants in the study were weighed with minimal clothing and without shoes on a digital scale (Seca, model 707, Germany). In order to calculate the body mass index (BMI) of the participants, the weight (kg) of the patients was divided by the square of their height (m2). Patients with a BMI between 25–29.9 Kg/m^2^ were considered overweight, and patients over 30 Kg/m^2^ were considered obese.

According to the guidelines of the World Health Organization, patients' blood pressure was measured with a standard manometer (mercury manometer RICHTER, Germany) after a 10‐min rest, and if it was abnormal, it was measured again after 1 h.

WHO Global Physical Activity Questionnaire (GPAQ) and Metabolic Equivalent (MET) were used to evaluate the amount and intensity of daily physical activity, respectively. A MET is defined as the amount of energy consumed while sitting (equivalent to 1 kcal/kg/h). Moderate to severe physical activity is defined as four times and eight times calorie intake compared to sitting quietly. The amount of activity more than 3000 MET and less than 1500 MET per week was considered intense and low physical activity, respectively.^8^

A trained interviewer asked participants about smoking, tobacco use and opium addiction. People who routinely smoked cigarettes during data collection were considered smokers. Also, people who continuously used tobacco products or opium during data collection were considered positive for tobacco use and opium addiction, respectively.

In addition to determining demographic features of the studied people, being informed of this laboratory finding and drug treatment in the case of pre‐notice, a sample of fasting blood was taken (after 12–14 h of fasting) to determine the level of serum uric acid and fasting plasma glucose (FPG) for each person.

FPG (KIMIA Kit, code 890410, Iran) and uric acid (measured by the colorimetric method.) were measured for all patients. In our study, the hyperuricaemia cut‐off was considered to be greater than the 75th percentile, which was 6 mg/dl in males and 5 mg/dl in females.

The senior researcher trained all the interviewees prior to data collection, and he also continuously evaluated the validity of the data collected. Although agreement indices were not measured in this study, careful quality control was performed to minimize errors.

SPSS software version 16 (SPSS Inc.) was used to analyse data. Categorical variables were reported as numbers and percentages, and a chi‐square test was used to compare them between the two groups. Continuous variables were reported as medians (with interquartile ranges [IQRs]) based on their abnormal distribution according to the Kolmogorov–Smirnov test, and the Mann–Whitney test was used to compare them between individuals with and without hyperuricaemia. *p* < .05 was considered as significant.

### 2.1 Sample size estimation

To determine the sample size based on the study of Zhu et al., the prevalence of hyperuricaemia was considered to be 21.4%, and the minimum required sample size was calculated using the following formula^9^ and considering Z = 1.96, *p* = .21, and D = 0.03:

$$n=\frac{Z^{2}P\left(1-P\right)}{d^{2}}=\frac{1.96^{2}0.21\left(1-0.21\right)}{0.03^{2}}=708$$

To increase the power of our regression, we investigated 1008 people in our study.

### 2.2 Statistical analysis

To determine the potential predictors of hyperuricaemia, univariable and multivariable survey logistic regression models were used. Variables significantly different in individuals with and without hyperuricaemia were included in the regression models, and the odds ratios (AOR) were reported.

## 3 RESULTS

A number of 1008 cases were entered into the study. The relationship between the studied variables and the prevalence of hyperuricaemia is shown in Table 1. According to the results of this study, 254 patients had uric acid levels above the 75th percentile (more than 6 mg/dl in males, and 5 mg/dl in females). No significant difference was seen between gender (*p* = .249) and age groups (*p* = .125) of people with and without hyperuricaemia. The prevalence of overweight/obesity was significantly higher in patients with hyperuricaemia (*p* < .001). The prevalence of low physical activity in patients with hyperuricaemia was significantly higher than in those with normal uric acid levels (*p* = .033). The prevalence of smoking (*p* = 406), tobacco consumption (*p* = .669) and opium addiction (*p* = .261) in people with and without hyperuricaemia did not show a significant difference. The frequency of hypertension was significantly higher in people with high uric acid levels compared to people with normal uric acid levels (31.9% and 22.8%, respectively; *p* = .004). The duration of hypertension was significantly higher in hyperuricaemic individuals (*p* = .022). The prevalence of diabetes (*p* = .794) and the duration of diabetes (*p* = .371) in people with and without hyperuricaemia did not show a significant difference. The duration of hypertension and diabetes was converted into categorized variables according to their median (48 and 72 months, respectively). The prevalence of a longer period of hypertension (more than 48 months) in people with hyperuricaemia was significantly higher than in those without hyperuricaemia (*p* = .025). The prevalence of a longer duration of diabetes (more than 72 months) between the two groups of people with and without hyperuricaemia was similar (*p* = .986).

**TABLE 1:** The relationship between the studied variables and the presence of hyperuricaemia

Variables	Total	Hyperuricaemia		*p*‐value
		No	Yes	
	*N* = 1008	*N* = 754	*N* = 254	
Gender, *n* (%)				
Man	441 (43.8)	322 (42.7)	119 (46.9)	0.249^a^
Woman	567 (56.3)	432 (57.3)	135 (53.1)	
Age groups, *n* (%)				
Under 20 years	18 (1.8)	15 (2.0)	3 (1.2)	0.125^a^
21 to 40 years	283 (28.1)	223 (29.6)	60 (23.6)	
41 to 60 years	455 (45.1)	340 (45.1)	115 (45.3)	
61 to 80 years	237 (23.5)	167 (22.1)	70 (27.6)	
Above 80 years	15 (1.5)	9 (1.2)	6 (2.4)	
Overweight/obesity, *n* (%)				
Yes (BMI ≥ 25 kg/m^2^)	676 (67.1)	467 (61.9)	209 (82.3)	<0.001^a^
No (BMI < 25 kg/m^2^)	332 (32.9)	287 (38.1)	45 (17.7)	
Physical activity, *n* (%)				
Low (<1500 MET)	538 (53.4)	387 (51.3)	151 (59.4)	0.033^a^
Moderate (1500–3000 MET)	364 (36.1)	279 (37.0)	85 (33.5)	
Intense (>3000 MET)	106 (10.5)	88 (11.7)	18 (7.1)	
Smoking, *n* (%)	84 (8.3)	66 (8.8)	18 (7.1)	0.406^a^
Tobacco, *n* (%)	57 (5.7)	44 (5.8)	13 (5.1)	0.669^a^
Opium addiction, *n* (%)	86 (8.5)	60 (8.0)	26 (10.2)	0.261^a^
Hypertension, *n* (%)	253 (25.1)	172 (22.8)	81 (31.9)	0.004^a^
Duration of hypertension, median (IQR), (months)	48.0 (24.0–120.0)	48.0 (24.0–96.0)	60.0 (33.0–144.0)	0.022^b^
Duration of hypertension, *n* (%)				
1–48 months	126 (49.8)	94 (54.7)	32 (39.5)	0.025^a^
49–480 months	127 (50.2)	78 (45.3)	49 (60.5)	
Diabetes, *n* (%)	164 (16.3)	124 (16.4)	40 (15.7)	0.794^a^
Duration of diabetes, median (IQR), (months)	72.0 (36.0–120.0)	72.0 (36.0–129.0)	72.0 (15.0–120.0)	0.371^b^
Duration of Diabetes, *n* (%)				
4–72 months	90 (54.9)	68 (54.8)	22 (55.0)	0.986^a^
73–360 months	74 (45.1)	56 (45.2)	18 (45.0)	

Abbreviations: BMI: Body mass index, MET: Metabolic Equivalent (The amount of energy consumed while sitting (equivalent to 1 kcal/kg/h)).

^a^ Chi‐square test.

^b^ Mann–Whitney test.

In order to determine the potential predictors of hyperuricaemia, univariable and multivariable survey logistic regression models were used on variables whose frequency was significantly different in individuals with and without hyperuricaemia (Table 2). Based on the univariate logistic regression model, the presence of overweight/obesity, low physical activity and hypertension increased the chances of hyperuricaemia in the subjects by 2.85, 1.91, compared to those with intense physical activity and 1.58, respectively (*p* < .001, *p* = .019 and *p* = .004, respectively). According to the results of a multivariate analysis on the three variables, the amount of physical activity did not have a significant effect on increasing the chances of hyperuricaemia and the two variables of overweight/obesity (OR = 2.67; 95% CI = 1.87–3.82) and hypertension (OR = 1.40; 95% CI = 1.02–1.93) significantly increased the chances of hyperuricaemia in the subjects.

**TABLE 2:** Univariate and multivariate logistic regression models for identifying determinants of hyperuricaemia

	Univariate		Multivariate	
	*p*‐value	OR (95% CI)	*p*‐value	OR (95% CI)
Overweight/obesity				
Yes (BMI ≥ 25 kg/m^2^)	<0.001	2.85 (2.0–4.07)	<0.001	2.67 (1.87–3.82)
No (BMI < 25 kg/m^2^)	‐	‐	‐	‐
Physical activity				
Low (<1500 MET)	0.019	1. 91 (1.11–3.28)	0.073	1.66 (0.95–2.88)
Moderate (1500–3000 MET)	0.165	1.49 (0.85–2.61)	0.317	1.34 (0.75–2.37)
Intense (>3000 MET)	‐	‐	‐	‐
Hypertension				
Yes	0.004	1.58 (1.16–2.17)	0.04	1.40 (1.02–1.93)
No	‐	‐	‐	‐

Abbreviations: BMI: Body mass index, MET: Metabolic Equivalent (The amount of energy consumed while sitting (equivalent to 1 kcal/kg/h)).

## 4 DISCUSSION

According to the results of our study, overweight/obesity and hypertension increase the risk of hyperuricaemia in the general population. Due to the increasing prevalence of hyperuricaemia, it should be considered as an important health issue. So far, several studies have emphasized the relationship between the presence of hyperuricaemia and cardiovascular risk factors, including hypertension and metabolic syndrome^10^, ^11^, ^12^ as in a recent cross‐sectional study; Yazdi et al.^13^ showed that the ratio of uric acid to high‐density lipoprotein (HDL) cholesterol is significantly higher in people with metabolic syndrome than in healthy individuals. Despite the similarity of the results of the present study with previous studies, there are also differences. Different study designs and different criteria for confirming hyperuricaemia in patients can be the main cause of these differences.

In previous studies, inflammatory response, oxidative stress, insulin resistance, endothelial dysfunction and endoplasmic reticulum stress have been mentioned as the main mechanisms of hyperuricaemia's effect on the development of cardiovascular diseases.^14^ In our study, hypertension was associated with increased uric acid levels in patients. In line with our study in Zhang et al.,^15^ a prospective cohort study of 6424 patients, hypertension was also associated with an increased risk of hyperuricaemia. In another cross‐sectional study of 3119 people over the age of 50 in a rural population in China, high blood pressure increased the risk of high uric acid levels.^10^ Although the mechanism of association between hyperuricaemia and hypertension is still unclear, according to the findings of in vitro studies, uric acid causes damage to endothelial cells, vasoconstriction and high blood pressure by inhibiting the release of nitric oxide from endothelial cells, activating the renin‐angiotensin system and oxidative stress.^16^ Chen et al.’s^17^ study of animal models and generally healthy early Parkinson's disease patients found no association between uric acid levels and blood pressure. Another possible cause of elevated uric acid in hypertensive patients is the use of certain drugs, including diuretics, β‐blockers, angiotensin‐converting enzyme inhibitors and non‐losartan angiotensin II receptor blockers.^18^ In our study, the duration of hypertension in patients with hyperuricaemia was significantly longer, which could be due to the long‐term use of antihypertensive drugs, so more studies should be performed on new cases of hypertension to study the relationship between hypertension and uric acid levels.

In our study, overweight/obesity was associated with hyperuricaemia. In a study by Choi et al.,^19^ BMI higher than 25 was significantly associated with an increased risk of gout in patients, and weight loss was a protective factor against gout. The link between obesity and hyperuricaemia can be due to insulin resistance caused by oxidative stress and inflammatory processes. Hyperinsulinaemia is associated with insulin resistance and increases the activity and expression of urate transporter protein 1 (URAT1) and, consequently, uric acid reabsorption through renal proximal tubular cells.^14^, ^20^ However, in our study, no association was found between hyperuricaemia and diabetes. In a study by Krishnan et al.,^5^ elevated serum urate levels were an independent risk factor for type 2 diabetes in non‐obese patients. In a study by Van der Schaft et al.,^6^ serum uric acid was associated with incidental prediabetes in patients with normal blood sugar but not with the development of type 2 diabetes in prediabetic subjects. In a study conducted on mice by Lu et al.,^21^ there was no association between high uric acid levels and diabetes, and they concluded that high uric acid levels did not cause diabetes but could accelerate it by damaging glucose tolerance.

In our study, although less physical activity significantly increased the risk of hyperuricaemia in univariate analysis, the relationship between physical activity and hyperuricaemia was not significant in multivariate analysis after adjustment of the effect of overweight/obesity and hypertension variables. Dong et al.,^22^ in a study of 38,855 rural China populations, showed that regular physical activity independently reduced serum uric acid levels and the prevalence of hyperuricaemia. However, in the regression analysis performed in the study of Dong et al.,^22^ adjustment was done for BMI but not for hypertension. In the study by Qi et al.,^10^ low physical activity in men was associated with an increased risk of high uric acid levels. It seems that racial differences in the relationship between physical activity and hyperuricaemia need to be further evaluated.

In this study, the prevalence of hyperuricaemia has no significant relationship with age groups, smoking, tobacco consumption and opium addiction. Despite our study, Hanna et al.^23^ showed that uric acid serum level is significantly lower in smoking people, but these people did not suffer from hyperuricaemia in our study. It was shown in another study conducted by Tomita et al.^24^ that, like our study, uric acid serum levels and hyperuricaemia in smoking people have no significant difference with people who have never smoked statistically. In this study, the majority of cases belonged to the age group of 41 to 60 (45.1%) years, which probably people aged 41–60 had a higher tendency to participate in the study due to their awareness of the benefits of the study.

One of the strengths of our study was its population‐based and appropriate sample size. The sampling method in our study was cluster. Among the advantages of this method is that it is economical in terms of cost and time compared to probability sampling methods. But the accuracy of this method is lower than simple random sampling. Our study also had limitations, including the fact that many factors, including smoking and the duration of hypertension, were self‐reported, and there was a possibility of bias in these variables. Another limitation of the study was the possible effects of the drugs used and their lack of evaluation, including diuretics, aspirin and dietary habits on patients' uric acid levels separately. Another limitation of our study was that, unfortunately, the dietary profile of the participants was not recorded at the time of sampling. Despite all the limitations mentioned, our findings are helpful in determining the appropriate strategy to identify people who are most at risk for hyperuricaemia for the prevention and early treatment of this disorder.

## 5 CONCLUSION

Our study showed that two independent factors for the development of hyperuricaemia are hypertension and overweight/obesity. Since hyperuricaemia is one of the influential factors in cardiovascular events, these events can be prevented by determining the appropriate strategy for early diagnosis and treatment of this metabolic disorder.

Considering that our study was conducted in an urban population and environmental and genetic factors can also be involved in the relationship between hyperuricaemia and other cardiovascular disease risk factors, cohort studies with a larger sample size are needed in this field.

## AUTHOR CONTRIBUTIONS

Farzaneh Yazdi, Mohammad Reza Shakibi, Hamid Najafipour, Amir Baniasad, Mohammad Javad Najafzadeh and Samira Sistani designed the study and collected data, Amir Baniasad, Mohammad Javad Najafzadeh wrote the first draft of the paper and submitted the manuscript, Fatemeh Yazdi contributed to writing and revision of the manuscript. All authors contributed to finalizing the manuscript.

## FUNDING INFORMATION

This research project was funded by the Kerman University of Medical Sciences.

## CONFLICT OF INTEREST

The authors declare that they have no conflict of interest.

## ETHIC STATEMENT

The study protocol was reviewed and approved by the ethics committee of Kerman University of Medical Sciences (ethic code: IR.KMU.REC.1394.59). Informed consent was obtained from all participants in the study.

## Data Availability

The data that support the findings of this study are available from the corresponding author upon reasonable request.
